# Genome-Wide DNA Methylation Changes Associated With High-Altitude Acclimatization During an Everest Base Camp Trek

**DOI:** 10.3389/fphys.2021.660906

**Published:** 2021-06-28

**Authors:** Ainash Childebayeva, Taylor Harman, Julien Weinstein, Trevor Day, Tom D. Brutsaert, Abigail W. Bigham

**Affiliations:** ^1^Department of Anthropology, University of Michigan, Ann Arbor, MI, United States; ^2^Department of Environmental Sciences, School of Public Health, Ann Arbor, MI, United States; ^3^Department of Archaeogenetics, Max Planck Institute for the Study of Human History, Jena, Germany; ^4^Department of Anthropology, Syracuse University, Syracuse, NY, United States; ^5^Department of Biology, Mount Royal University, Calgary, AB, Canada; ^6^Department of Exercise Science, Syracuse University, Syracuse, NY, United States; ^7^Department of Anthropology, University of California, Los Angeles, Los Angeles, CA, United States

**Keywords:** epigenetics (DNA methylation), genome-wide DNA methylation analysis, high altitude acclimatization, HIF pathway, hypoxia

## Abstract

The individual physiological response to high-altitude hypoxia involves both genetic and non-genetic factors, including epigenetic modifications. Epigenetic changes in hypoxia factor pathway (HIF) genes are associated with high-altitude acclimatization. However, genome-wide epigenetic changes that are associated with short-term hypoxia exposure remain largely unknown. We collected a series of DNA samples from 15 participants of European ancestry trekking to Everest Base Camp to identify DNA methylation changes associated with incremental altitude ascent. We determined genome-wide DNA methylation levels using the Illumina MethylationEPIC chip comparing two altitudes: baseline 1,400 m (day 0) and elevation 4,240 m (day 7). The results of our epigenome-wide association study revealed 2,873 significant differentially methylated positions (DMPs) and 361 significant differentially methylated regions (DMRs), including significant positions and regions in hypoxia inducible factor (HIF) and the renin–angiotensin system (RAS) pathways. Our pathway enrichment analysis identified 95 significant pathways including regulation of glycolytic process (GO:0006110), regulation of hematopoietic stem cell differentiation (GO:1902036), and regulation of angiogenesis (GO:0045765). Lastly, we identified an association between the *ACE* gene insertion/deletion (I/D) polymorphism and oxygen saturation, as well as average *ACE* methylation. These findings shed light on the genes and pathways experiencing the most epigenetic change associated with short-term exposure to hypoxia.

## Introduction

Altitude acclimatization in humans is characterized by complex physiological responses, which include the cardiovascular, hemopoietic, respiratory, and metabolic systems [for review, see Palmer (2010)]. Each system responds uniquely to low oxygen environments. For example, cardiovascular output increases (i.e., increased heart rate and stroke volume) upon initial altitude exposure and returns to pre-altitude baseline after several days of acclimatization [for review, see Naeije (2010)]. The respiratory system’s response is to initiate hyperventilation. The hypoxic ventilatory response (HVR) is elicited shortly upon exposure to high altitude, with ventilatory acclimatization emerging following 5–7 days of sustained exposure to hypoxia (Powell et al., 1998). Lastly, the hemopoietic response in the form of increased erythrocyte production is evident after several days to weeks of exposure (Rodriguez et al., 2000). Each of these responses facilitates acute acclimatization to the low ambient oxygen tension present at high altitudes, allowing humans to acclimatize to hypoxic conditions.

Epigenetic change is one mechanism through which physiological acclimatization may occur. Epigenetic modifications can affect gene expression and include DNA methylation, histone tail modifications, and short RNA regulation. The most well-studied epigenetic mark is DNA methylation, the addition of a methyl group primarily to cytosine bases. DNA methylation patterns can change upon exposure to various environmental conditions, including exposure to different diets, stress, and toxicants (Dolinoy et al., 2007; Baccarelli et al., 2009; Colacino et al., 2012; Childebayeva et al., 2019b). Previous studies have demonstrated that changes in DNA methylation are associated with exposure to the low oxygen environment of high altitude (Alkorta-Aranburu et al., 2012; Childebayeva et al., 2019a, b, 2020). These studies show that genes in the hypoxia inducible factor (HIF) pathway exhibit changes in DNA methylation associated with high-altitude exposure.

The HIF pathway is the main oxygen sensing pathway that regulates cellular homeostasis in metazoans (Bigham and Lee, 2014). The pathway takes its name after the master transcriptional regulator HIF, a heterodimeric transcription factor that is formed by one of three α-subunits (HIF-1α, HIF-2α, or HIF-3α) and a β-subunit (also known as ARNT). In normoxia, HIF1A is hydroxylated and subsequently degraded by the ubiquitin–proteosome pathway. Under hypoxia, this hydroxylation is inhibited by the lack of oxygen availability, leading to the dimerization of HIF and activation of target genes. HIF is responsible for transducing changes in oxygen tension to changes in gene expression through hypoxia response elements (HREs) (Wang and Semenza, 1995; Kaelin and Ratcliffe, 2008; Semenza, 2012). The renin–angiotensin system or RAS is a second pathway that is involved in the response to hypoxia. It is one of the body’s most important regulators of blood pressure and inflammation (Muller et al., 1997; Rupert et al., 2003). The RAS protein, angiotensin converting enzyme (ACE), is a central peptide in blood-pressure regulation responsible for converting angiotensin-I to the vasoconstrictor, angiotensin-II. An insertion/deletion (I/D) polymorphism in ACE is associated with physical performance at high altitude (Woods et al., 2002; Tsianos et al., 2005). The I-allele has been associated with higher levels of submaximal oxygen saturation (SaO_2_) among Andean Quechua (Bigham et al., 2008), and in trekkers of European ancestry (Woods et al., 2002).

Previous research by our group has shown that acclimatization to hypoxia is associated with DNA methylation changes in HIF pathway genes including *EPAS1*, *EPO*, *PPARa*, and *RXRA* (Childebayeva et al., 2019a). However, it is not well understood what other genes and pathways display DNA methylation changes upon exposure to hypoxia. To understand how acclimatization to hypoxia affects genome-wide DNA methylation patterns, we performed an epigenome-wide association study in individuals trekking to Everest Base Camp. Our analysis compared baseline methylation measured in Kathmandu, Nepal at 1,400 m (day 0) with methylation measured at a high-altitude location, Pheriche, Nepal at 4,240 m (day 7 of the trek).

## Materials and Methods

### Ethics Statement

Ethical approval was received from the Syracuse University Institutional Review Board (Protocol 18-006) and the University of Michigan Institutional Review Board (HUM00141118). The study abided by the Canadian Government Tri-Council policy on research ethics with human participants (T2) and the Declaration of Helsinki, except for registration in a database. Ethical approval was received also from the Mount Royal University Human Research Ethics Board (Protocol 100012 and 101361) and harmonized with the Nepal Health Research Council (Protocol 109-2017).

### Study Design and Sample Collection

**FIGURE 1:**
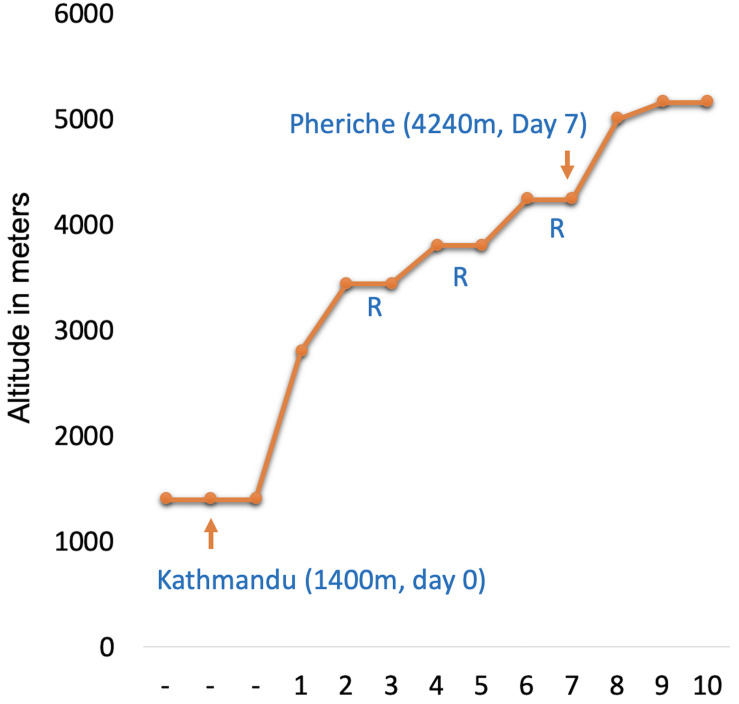
Ascent profile with sample collection altitudes indicated with arrows and labels. Study participants flew from baseline (day 0) Kathmandu to 2,800 m to begin the trek. Three non-trekking rest days are indicated by “R.” Epigenome wide association study was performed on matched samples collected at 1,400 m (day 0) and 4,240 m (day 7).

Thirty-two samples (16 samples at 1,400 m and 16 samples at 4,240 m) corresponding to 16 unique individuals were selected from a larger participant cohort from the research expedition to Everest Base Camp in the Nepal Himalaya (Childebayeva et al., 2019a). Briefly, study participants and researchers flew from Kathmandu (baseline) to Lukla from where the research group trekked for 10 days from 2,800 to 5,160 m (Figure 1). In the morning between 06:00 and 08:00 local time at 1,400 m (Kathmandu; day 0) and 4,240 m (Pheriche; day 7), saliva samples for DNA and physiological measures were taken following one night of sleep at each altitude. Physiological measurements included hemoglobin concentration [Hb], patient end-tidal carbon dioxide (P_ET_CO_2_), a measure of CO_2_ partial pressure in expired air, which reflects the CO_2_ level in the arterial blood, and peripheral oxygen saturation (SaO_2_). Detailed information on phenotype collection and sampling is provided in Childebayeva et al. (2019a). All participants were healthy, non-pregnant, non-lactating, non-smokers between 19 and 41 years of age. All participants were of self-reported European ancestry and had at least 1 year since their last altitude experience. Participant characteristics can be found in Table 1.

**TABLE 1:** Participant characteristics.

	1,400 m (day 0)	4,240 m (day 7)
Hemoglobin (mg/L)*	13.1 (1.8)	14.8 (1.4)
BMI (kg/m^2^)^#^	22.6 (2.4)	22.4 (2.2)
P_ET_CO_2_ (Torr)**	30.7 (3.2)	22.1 (2.9)
SaO_2_ (%)**	97.1 (1.1)	89.8 (2.4)
% Female	53%	
Age, year	23.6 (6.0)	

Data are means (SD). Significance symbols denote the difference between Kathmandu baseline and each altitude. ^#^p-value < 0.10; *p-value < 0.01; and **p-value < 0.001.

### Phenotype Testing

We performed linear mixed models using the R package lmerTest to test for significant differences in phenotypes between Kathmandu and Pheriche (Table 1). The following model was tested: Phenotype ∼ Altitude + Sex + Age + (1 | ID).

### DNA Methylation

We generated DNA methylation data for ∼850,000 CpG sites using the Illumina Infinium^®^ MethylationEPIC BeadChip assay for 32 samples in our study. We used the EZ-96 DNA Methylation^TM^ Kit (Zymo Research, Irvine, CA, United States) to bisulfite convert each DNA sample following the standard protocol with alternative incubation conditions optimized for the Illumina Infinium^®^ MethylationEPIC BeadChip assay. We used R for data processing and analysis implementing the packages minfi, ChAMP, and SmartSVA (Aryee et al., 2014; Morris et al., 2014; Chen et al., 2017). Based on QC metrics, two samples from the same participant failed and were excluded from all analyses; thus, the final sample size was *n* = 30 (15 at 1,400 m and 15 at 4,240 m).

Data normalization was performed using the funnorm normalization function in minfi (Aryee et al., 2014). We removed all probes that were above the 10e5 detection *p*-value threshold (*N* = 8,126) in more than 5% of the samples, all cross-reactive probes, probes associated with sex chromosomes, probes containing SNPs with MAF > 5% at target CpG sites, single base extension sites of type I probes, and in the body of the probe (Chen et al., 2013). All analyses were performed with *N* = 657,569 sites after normalization and probe removal. Samples were tested for batch effects using singular value decomposition (SVD) analysis in champ. SmartSVA (Chen et al., 2017) was used to perform a surrogate variable test, and the surrogate variable 1 was used for correcting for any saliva cell type differences associated with altitude. SmartSVA is a surrogate variable analysis method that can be used for reference-free adjustment for cell mixtures (Chen et al., 2017).

### Differential Methylation Testing

Fully processed M-values were tested for differential methylation using the package limma (Ritchie et al., 2015). The following model was used to test for the differentially methylated positions (DMPs): DNA methylation ∼ Sample ID + Altitude + Surrogate Variable 1 (from smartSVA). *P*-values were adjusted for multiple testing using the false-discovery rate (FDR) following the Benjamini–Hochberg procedure (Hochberg and Benjamini, 1990) to produce FDR-corrected q-values. Differentially methylated regions (DMRs) were determined using DMRcate with default parameters (lambda = 1,000, *C* = 2, min.CpG sites = 2) (Peters et al., 2015). Pathway enrichment was performed using the package methylGSA (Ren and Kuan, 2019).

Angiotensin converting enzyme genotyping was performed using the same protocol as in Bigham et al. (2008). We extracted *ACE* CpG sites from the MethylationEPIC array to assess its methylation status independent from the epigenome-wide association analysis. We tested the relationship between ACE I/D status and SaO_2_ separately for Kathmandu and Pheriche using linear modeling and adjusting for age and sex. The relationship between *ACE* genotype and phenotypes, as well as *ACE* genotype and *ACE* DNA methylation, was tested using linear mixed modeling in R using the lmerTest package (Kuznetsova et al., 2017). The linear mixed model was adjusted for altitude, age, sex, and individual IDs. Plotting was performed using the ggplot2 package (Wickham, 2009).

## Results

### Participant Demographics

Our study group included *n* = 15 participants of self-reported European ancestry, with 53% females and the average BMI of 22.60 (SD 2.36) at baseline. Participant characteristics can be found in Table 1.

### Physiological Changes With Altitude Exposure

We detected significant physiological changes between altitude 1,400 m (day 0) and 4,240 m (day 7) (henceforth physiological variables are referred to as phenotypes in this manuscript) in arterial oxygen saturation (SaO_2_), hemoglobin concentration [(Hb)], and end-tidal carbon dioxide partial pressure (P_ET_CO_2_) (Table 1). Briefly, we observed a significant increase in [Hb] and a significant decrease in SaO_2_ and P_ET_CO_2_ with increasing altitude. The physiological responses we have reported are expected at high altitude, i.e., lower arterial oxygen saturation due to decreased ambient PO_2_, a decrease in P_ET_CO_2_ indicating an increase in alveolar ventilation, and higher [Hb], reflecting the body’s physiological response to low-oxygen conditions by increasing hemoglobin production.

### Differential Methylation Analysis

We generated DNA methylation data for ∼850,000 CpG sites using the Illumina Infinium^®^ MethylationEPIC BeadChip. After QC, we performed differential methylation analysis on 755,636 probes. We identified 2,873 DMPs at q-value < 0.10 (Supplementary Table 1) that differed between baseline 1,400 and 4,240 m genome-wide inflation factor λ = 1.2. Among these, we identified HIF pathway genes: *ANGPT1, CREBBP, CUL2*, *HIF1A*, *HK1*, *HMOX1*, *PDK1* (two significant CpG sites), *PIK3R3*, *PLCG1*, *PRKCG*, *RELA*, and *STAT3*, and RAS pathway genes: *ABL1*, *ANGPT1*, *EFNA3*, *FGFR1*, *GAB1*, *GNB1*, *GNB3*, *GNB4*, *GRB2*, *KITLG*, *KRAS*, *MAPK10*, *PAK1*, *PAK2*, *PDGFA*, *PIK3R3*, *PLCG1*, *PRKCG*, *PTPN11*, *RALA*, *RAP1A*, *RAP1B*, *RASA3* (five significant CpG sites), *RASSF1*, *RELA*, *RGL2*, and *RIN1* (Table 2). We also identified genes associated with inflammation: *IL12B*, *TRIM31, NLRP3, IL1RAP*, among others, and genes associated with cognitive function: *ASH1L* and *TNIK.*

**TABLE 2:** Significant CpG sites associated with HIF and RAS pathways.

Pathway	Gene	CpG	*p*-value	*q*-value	Chr	Position (hg19)	Relation to island
HIF	*ANGPT1*	cg09443479	1.96E-04	0.08	8	108,511,174	OpenSea
	*CREBBP*	cg16560077	7.48E-05	0.05	16	3,781,408	Island
	*CUL2*	cg09080721	1.76E-04	0.07	10	35,361,575	OpenSea
	*HIF1A*	cg16788202	2.45E-04	0.08	14	62,162,340	Island
	*HK1*	cg06506461	3.14E-04	0.09	10	71,112,319	OpenSea
	*HMOX1*	cg15724965	1.87E-05	0.03	22	35,777,001	Island
	*PDK1*	cg13462525	7.98E-05	0.05	2	173,420,046	N_Shore
	*PDK1*	cg11703569	4.63E-05	0.04	2	173,421,320	Island
	*PIK3R3*	cg12800095	9.33E-05	0.06	1	46,594,087	OpenSea
	*PLCG1*	cg13312309	5.68E-05	0.05	20	39,799,964	OpenSea
	*PRKCG*	cg14975881	3.28E-05	0.04	19	54,389,945	N_Shelf
	*RELA*	cg04962756	2.35E-06	0.01	11	65,425,928	OpenSea
	*STAT3*	cg09804439	1.59E-04	0.07	17	40,540,457	Island
RAS	*ABL1*	cg13609937	4.40E-05	0.04	9	133,588,314	Island
	*ANGPT1*	cg09443479	1.96E-04	0.08	8	108,511,174	OpenSea
	*EFNA3*	cg06058618	6.65E-06	0.02	1	155,057,452	Island
	*FGFR1*	cg00676030	1.74E-05	0.03	8	38,307,962	OpenSea
	*GAB1*	cg24244452	3.54E-04	0.09	4	144,284,260	OpenSea
	*GNB1*	cg14953148	1.56E-04	0.07	1	1,792,846	OpenSea
	*GNB3*	cg06444189	2.38E-05	0.03	12	6,953,740	OpenSea
	*GNB4*	cg12872693	4.08E-04	0.10	3	179,168,798	Island
	*GRB2*	cg11495544	3.76E-04	0.10	17	73,402,155	S_Shore
	*KITLG*	cg22688836	6.67E-05	0.05	12	88,967,594	OpenSea
	*KRAS*	cg02850821	8.03E-06	0.02	12	25,403,680	OpenSea
	*MAPK10*	cg03886687	2.43E-04	0.08	4	87,281,409	OpenSea
	*PAK1*	cg26996201	2.86E-04	0.09	11	77,122,864	Island
	*PAK2*	cg02319016	1.34E-06	0.01	3	196,469,777	S_Shelf
	*PDGFA*	cg22466784	2.41E-04	0.08	7	540,176	OpenSea
	*PIK3R3*	cg12800095	9.33E-05	0.06	1	46,594,087	OpenSea
	*PLCG1*	cg13312309	5.68E-05	0.05	20	39,799,964	OpenSea
	*PRKCG*	cg14975881	3.28E-05	0.04	19	54,389,945	N_Shelf
	*PTPN11*	cg16207631	2.76E-04	0.09	12	112,856,603	Island
	*RALA*	cg19104112	2.75E-04	0.09	7	39,663,043	Island
	*RAP1A*	cg25355888	2.83E-04	0.09	1	112,162,642	Island
	*RAP1B*	cg00758412	2.23E-04	0.08	12	69,033,023	OpenSea
	*RASA3*	cg21364828	1.18E-04	0.06	13	114,825,608	OpenSea
	*RASA3*	cg13818243	2.76E-04	0.09	13	114,789,734	S_Shelf
	*RASA3*	cg04421280	1.20E-04	0.06	13	114,898,225	Island
	*RASA3*	cg00427150	1.92E-04	0.07	13	114,770,568	N_Shelf
	*RASA3*	cg20028528	2.20E-04	0.08	13	114,812,184	N_Shore
	*RASSF1*	cg25486143	3.20E-04	0.09	3	50,378,527	Island
	*RELA*	cg04962756	2.35E-06	0.01	11	65,425,928	OpenSea
	*RGL2*	cg08312215	4.75E-05	0.04	6	33,266,943	Island
	*RIN1*	cg15082918	2.81E-04	0.09	11	66,104,153	S_Shore

In order to detect biological pathways overrepresented among the significant CpG sites from the analysis of differential methylation, we performed a pathway enrichment analysis using the methylgometh function in the R package methylGSA (Ren and Kuan, 2019). Ninety-five significant pathways were identified by methylgometh including the GO pathways regulation of glycolytic process (GO:0006110), regulation of hematopoietic stem cell differentiation (GO:1902036), and regulation of angiogenesis (GO:0045765) (Supplementary Table 2). Other pathways of interest included brain development (GO:0007420), negative regulation of neuron differentiation (GO:0045665), and interleukin-1-mediated signaling pathway (GO:0070498).

We then tested for DMRs, i.e., contiguous regions in the genome that show differential methylation between phenotypes or groups. We used DMRcate (Peters et al., 2015) to find DMRs between low- (1,400 m) and high-altitude (4,240 m) samples. Using this approach, we identified 361 significant DMRs out of 657,408 possible DMRs (Supplementary Table 3). These included DMRs near/in genes associated with the HIF pathway: *HIF1A* and *ENO1* (glycolytic enzyme), and the RAS pathway: *ABL1*, *FGFR3*, *KRAS*, *RASA3*, and *RGL2*.

### Phenotype Associations

To determine if changes in DNA methylation could be driving acclimatization, we performed association testing between significant genome-wide methylation positions and phenotypes associated with high-altitude acclimatization. To do so, we focused our analysis on significant CpG sites identified in the DMP analysis (*N* = 2,873) and phenotypes that were significantly different between the groups (Table 1) including SaO_2_, [Hb], and P_ET_CO_2_. Two CpG sites, cg16546681 (chr1:155244518, *q*-value = 0.01, β regression coefficient = 6.46) in the gene *CLK2* and cg14548038 (chr9:140178418, *q*-value = 0.03, β regression coefficient = 4.73) upstream of the gene *TOR4A*, were significantly positively associated with SaO_2_ (%). No significant associations were identified for [Hb] or P_ET_CO_2_ after correcting for multiple comparisons.

### ACE I/D, Oxygen Saturation, and DNA Methylation

**FIGURE 2:**
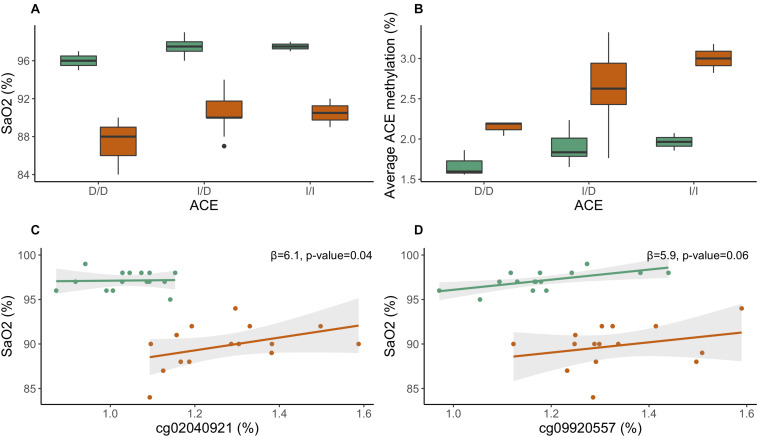
Relationship between ACE I/D, ACE methylation, and SaO_2_. **(A)** Boxplot of arterial oxygen saturation by ACE I/D genotype. **(B)** Boxplot of average ACE DNA methylation by ACE I/D genotypes. **(C)** ACE CpG site cg02040921 plotted against SaO_2_. **(D)** ACE CpG site cg09920557 plotted against SaO_2_. Day 0: 1,400 m (K) is indicated in green and day 7: 4,240 m (P) is noted in orange.

We tested the relationship between *ACE*, a gene associated with high-altitude performance, and high-altitude phenotypes [SaO_2_, P_ET_CO_2_, (Hb)]. Individuals in this study were genotyped for the *ACE* I/D (rs4646994) polymorphism. We performed a genotypic test, wherein I/I and I/D genotypes were compared to D/D genotype, and identified a significant association between *ACE* genotype and SaO_2_. Individuals with genotypes I/D (β regression coefficient = 1.69, *p*-value < 0.01) and I/I (β regression coefficient = 1.85, *p*-value < 0.05) had significantly higher SaO_2_ than individuals with the D/D genotype at 1,400 m (Kathmandu); the relationship was not significant for 4,240 m (Pheriche) (Figure 2). In an additive model, the I-allele was associated with increased SaO_2_ (β regression coefficient = 1.03, *p*-value < 0.05) at 1,400 m; the relationship was also not significant for 4,240 m. In a dominant model, individuals who were either heterozygotes or homozygotes for the I-allele (grouped together) displayed higher SaO_2_ (β regression coefficient = 1.71, *p*-value < 0.01) at 1,400 m; the relationship was approaching significance (β regression coefficient = 2.79, *p*-value = 0.09) for 4,240 m. Our results suggest that the dominant model, wherein individuals carrying either the I/D or I/I alleles have higher oxygen saturation than individuals carrying the D/D allele, is best suited to explain the relationship between SaO_2_ ACE I/D in our study.

Average *ACE* methylation was positively associated with the I-allele when we tested the relationship using an additive model (β regression coefficient = 0.31, *p*-value = 0.03) (Figure 2B). We also tested the relationship between individual *ACE* CpG sites and high-altitude phenotypes. *ACE* CpG sites, cg02040921 and cg09920557, were associated with SaO_2_ (cg02040921: *p*-value = 0.04; cg09920557: *p*-value = 0.06). Increased methylation of *ACE* CpGs was associated with increased SaO_2_ (Figures 2C,D). No significant associations were identified for [Hb] or P_ET_CO_2_.

## Discussion

The role of epigenetic change, including DNA methylation, in acclimatization to short-term hypoxia exposure is not well characterized. We aimed to fill this gap using genome-wide DNA methylation data from the same individuals measured at different altitudes during a trek to Everest Base Camp. We identified significant associations between genome-wide DNA methylation and short-term altitude exposure, among which were CpG sites and regions associated with HIF pathway, including *HIF1A*, and RAS pathway genes.

We identified both a significant CpG position (DMP) and a DMR associated with hypoxia inducible factor 1A or *HIF1A*, which is a central gene in the body’s hypoxic response (Slemc and Kunej, 2016). In normoxia, HIF1A is degraded *via* ubiquitination but is allowed to accumulate in hypoxic conditions. This allows its protein product to bind to a constitutively expressed HIF1B forming a heterodimer that activates downstream genes (Wenger, 2002). HIF1A activity is under epigenetic control in human cancer cells and hematopoietic cell lines (Walczak-Drzewiecka et al., 2010; Nguyen et al., 2013; Cimmino et al., 2019). Importantly, the *HIF1A*-associated DMR identified here overlaps with the promoter region of the gene, suggesting that methylation at this locus may be associated with changes in gene expression.

We found significant CpG sites associated with the RAS pathway, including ones in the genes *ANGPT1* and *RASA3* (RAS P21 protein activator 3). Angiopoietins 1 and 2 are regulated by HIF1, and *ANGPT1* expression is associated with increased number of vessels without excessive permeability (Kelly et al., 2003). *ANGPT1* can be activated and repressed by HIF1 in a cell-specific manner (Kelly et al., 2003). *RASA3* (RAS P21 protein activator 3) is a Ras-GTPase activating protein that causes anemia and thrombocytopenia in mice when mutated (Blanc et al., 2012). RAS pathway is another canonical hypoxia-induced pathway. RAS has been linked to blood pressure (Fontes et al., 1994), cardiovascular disease (Lee et al., 1993), and primary hypertension (Frossard et al., 1998). The role of RAS in hypoxia has been explored in association with high-altitude pulmonary edema *via* the regulation of the pulmonary vascular tone (Stobdan et al., 2011).

We also found significant DNA methylation changes in genes outside of canonical pathways implicated in high-altitude acclimatization (i.e., HIF and RAS), including significant DNA methylation changes in genes associated with cognitive impairment [*ASH1L* (de Ligt et al., 2012; Crawley et al., 2016; Xi et al., 2020) and *TNIK* (Coba et al., 2012; Anazi et al., 2016)]. Cognitive decline is a common side effect of high-altitude hypoxia (Regard et al., 1989; Yan, 2014; Gao et al., 2015) that becomes apparent 1–2 weeks after initial exposure (Bolmont et al., 2000) and may improve to some degree upon acclimatization (Heinrich et al., 2019). This timing of the cognitive decline is consistent with our study design wherein we identified methylation changes in *ASH1L* and *TNIK* after 1 week of high-altitude exposure. In addition to methylation differences in genes associated with cognitive function, we also identified changes in several genes associated with inflammation. These included CpG sites in the genes *IL12B* (Glas et al., 2012; Liu et al., 2012) and *TRIM31* (Song et al., 2016; Wang et al., 2018).

We specifically focused on the RAS pathway gene *ACE* as it is centrally involved in circulatory homeostasis, and the *ACE* I/D polymorphism has been linked to endurance performance (Myerson et al., 1999), adaptation of highland resident/native populations (Qadar Pasha et al., 2001; Bigham et al., 2008), and performance at altitude (all those other citations). The *ACE* I allele is associated with lower ACE activity (Costerousse et al., 1993) and higher SaO_2_ (Woods et al., 2002), potentially as a result of an increased HVR (Patel et al., 2003). We identified an association between *ACE* genotypes I/D and I/I with higher SaO_2_, which is consistent with previous research showing a significant relationship between *ACE* and SaO_2_ (Woods et al., 2002; Bigham et al., 2008).

We found that the *ACE* I-allele was associated with higher average *ACE* methylation, which has been shown before in a study of birth weight and *ACE* (Rangel et al., 2014). Notably, the *ACE* I-allele is associated with lower serum and tissue ACE activity (Rigat et al., 1990; Costerousse et al., 1993; Woods et al., 2000). Since methylation is commonly associated with gene silencing, the association between *ACE* I-allele and higher DNA methylation suggests that *ACE* methylation may be involved in mediating decreased *ACE* expression in individuals with the I-allele.

Individuals at high altitude displayed increased [Hb] and decreased SaO_2_ and P_ET_CO_2_ compared to low altitude. We found two CpG sites, in the gene *CLK2* and near the gene *TOR4*, that were associated with SaO2. CDC like kinase 2 or CLK2 suppresses *PPARGC1A* transcriptional activity on gluconeogenic genes (Sahu et al., 2019) and thus downregulates hepatic gluconeogenesis and glucose output. We found *CLK2* methylation to be positively associated with SaO_2_, suggesting that *CLK2* expression is potentially decreased in hypoxic conditions, given methylation is linked to gene repression. Interestingly, the CpG site in *CLK2* is upstream of the gene *PKLR* that is significantly differentially methylated in high- compared to low-altitude Quechua (Childebayeva et al., 2020). We also found a CpG site upstream of *TOR4A* (Torsin family 4 member A), which is associated with dystonia (Cascalho et al., 2017). Dystonia is linked to hypoxic exposure, more specifically cerebral anoxia/hypoxia (Kuoppamaki et al., 2002; Kern et al., 2016), and our finding might indicate a potential epigenetic mechanism playing role in the development of this condition.

Tissue types can show different methylation profiles across the body, and the degree to which they correlate varies by study design, type of sample, or age (Langie et al., 2017). For example, there is evidence of a low correlation between salivary and blood global DNA methylation (Godderis et al., 2015). Here, we analyzed saliva. Saliva is an attractive tissue for the analysis of DNA methylation in field studies given its relative ease of collection compared to blood or other tissues (Langie et al., 2017). By focusing on a singular tissue type, our results may be restricted to salivary tissue alone. However, salivary DNA methylation patterns have been shown to correlate with DNA methylation from blood (Thompson et al., 2013; Langie et al., 2016), intestinal mucosa (Hearn et al., 2019), and the brain (Smith et al., 2015). Furthermore, saliva panels have shown proteomic changes upon hypoxic exposure in cell cultures (Jain et al., 2020), suggesting the relevance of this tissue for analyzing the overall hypoxic response. Therefore, we suggest that our analysis of saliva is an important first step in identifying DNA methylation changes to acute hypoxia that may be relevant to other bodily tissues.

Overall, our data demonstrate that various pathways and systems are affected by exposure to high altitude, including the HIF pathway, RAS pathway, cognitive performance, and inflammatory systems. Moreover, we identified a significant association between SaO_2_ and *ACE* I/D, and associations between *ACE* I/D and *ACE* methylation, further highlighting the connection between *ACE* and SaO_2_ as well as the role of *ACE* in altitude acclimatization.

## Data Availability Statement

The data that support the findings of this study are available from the corresponding author AC, upon request.

## Ethics Statement

The studies involving human participants were reviewed and approved by Syracuse University Institutional Review Board (Protocol 18-006), University of Michigan Institutional Review Board (HUM00141118), Mount Royal University Human Research Ethics Board (Protocol 100012 and 101361), and Nepal Health Research Council (Protocol 109-2017). The patients/participants provided their written informed consent to participate in this study.

## Author Contributions

AC, TB, and AB: conceptualization. AC, TD, and JW: data curation. AC: formal analysis. TD and AC: funding acquisition. AC and TH: investigation. AC and AB: writing—original draft preparation. AB, AC, TB, TD, and TH: writing—review and editing. All authors contributed to the article and approved the submitted version.

## Conflict of Interest

The authors declare that the research was conducted in the absence of any commercial or financial relationships that could be construed as a potential conflict of interest.
